# The Antihypertensive Effects and Potential Molecular Mechanism of Microalgal Angiotensin I-Converting Enzyme Inhibitor-Like Peptides: A Mini Review

**DOI:** 10.3390/ijms22084068

**Published:** 2021-04-15

**Authors:** Qichen Jiang, Qi Chen, Tongqing Zhang, Meng Liu, Shunshan Duan, Xian Sun

**Affiliations:** 1 Freshwater Fisheries Research Institute of Jiangsu Province, 79 Chating East Street, Nanjing 210017, China; qichenjiang@live.cn (Q.J.); zhtq3@126.com (T.Z.); 2 Department of Ecology, Jinan University, Guangzhou 510632, China; cq92088@outlook.com (Q.C.); tssduan@jnu.edu.cn (S.D.); 3 Guangdong Center for Marine Development Research, Guangzhou 510220, China; 4 Key Laboratory of Bio-Resources and Eco-Environment of Ministry of Education, College of Life Sciences, Sichuan University, Chengdu 610065, China; liumengpro2015@outlook.com; 5 Zhuhai Key Laboratory of Marine Bioresources and Environment, Guangdong Provincial Key Laboratory of Marine Resources and Coastal Engineering, School of Marine Sciences, Sun Yat-Sen University, Guangzhou 510275, China; 6 Southern Marine Science and Engineering Guangdong Laboratory (Zhuhai), Zhuhai 519080, China

**Keywords:** antihypertensive effect, microalgae, ACE inhibitor, peptides, molecular docking

## Abstract

Hypertension causes many deaths worldwide and has shown an increasing trend as a severe non-communicable disease. Conventional antihypertensive drugs inevitably cause side effects, and great efforts have been made to exploit healthier and more-available substitutes. Microalgae have shown great potential in this regard and have been applied in the food and pharmaceutical industries. Some compounds in microalgae have been proven to have antihypertensive effects. Among these natural compounds, peptides from microalgae are promising angiotensin-converting enzyme (ACE) inhibitors because an increasing number of peptides show hypertensive effects and ACE inhibitory-like activity. In addition to acting as ACE inhibitors for the treatment of hypertension, these peptides have other probiotic properties, such as antioxidant and anti-inflammatory properties, that are important for the prevention and treatment of hypertension. Numerous studies have revealed the important bioactivities of ACE inhibitors and their mechanisms. This review discusses the antihypertensive effects, structure-activity relationships, molecular docking studies, interaction mechanisms, and other probiotic properties of microalgal ACE inhibitory peptides according to the current research related to microalgae as potential antihypertensive drugs. Possible research directions are proposed. This review contributes to a more comprehensive understanding of microalgal antihypertensive peptides.

## 1. Introduction

Microalgae are the main contributors to primary production in aquatic ecosystems. They are widespread, fast-growing, and resilient in different harsh environments, such as those with high salinity and temperature, as well as wastewater. In recent years, microalgae and their derived natural compounds have been regarded as important and sustainable food supplements and biodiesel sources for the purpose of overcoming food shortages and energy crises associated with population growth and limited land resources [1,2,3]. Furthermore, microalgae species are rich in bioactive compounds such as the primary nutrients carbohydrates, proteins, lipids, and probiotic materials such as vitamins and microelements [4,5,6,7,8,9,10,11,12]. In addition to their high production of nutrients, researchers have also recently found that an increasing number of bioactive compounds from microalgae show antioxidant [13,14], anticancer [15,16], anti-inflammatory [17,18], antimicrobial [19,20], and antiaging [21] properties with potential for the treatment and prevention of chronic diseases and their syndromes [22,23,24].

Hypertension, a chronic disease in humans, is a multifactorial disorder that is considered one of the major causes of premature death worldwide. As a “silent killer”, this chronic disease causes over nine million deaths annually and affects approximately one billion people [25]. Hypertension is a key risk factor for inducing cardiovascular diseases (CVDs), and the contribution of hypertension to CVD mortality has increased in some countries during the last two decades, especially in certain low-income countries that have the highest prevalence of hypertension [26]. Long-term hypertension also increases the risks of fatal conditions or events such as myocardial infarction [27], stroke [28], and kidney failure [29]. The pathogenesis of hypertension is complicated and unclear, and many factors, such as body mass index, sex, insulin resistance, high alcohol and salt intake, low potassium and calcium intake, stress, aging, and a sedentary lifestyle, are involved [30]. It should also be noted that free radical formation mediates some of the effects of hypertension. For instance, the proinflammatory actions caused by hypertension could increase the formation of hydrogen peroxide and free radicals, such as superoxide anions and hydroxyl radicals, in plasma to finally reduce the formation of nitric oxide by the endothelium [31]. The imbalance in the production and function of endothelial factors, such as nitric oxide, is associated with vascular physiological function [32].

**Figure 1:**
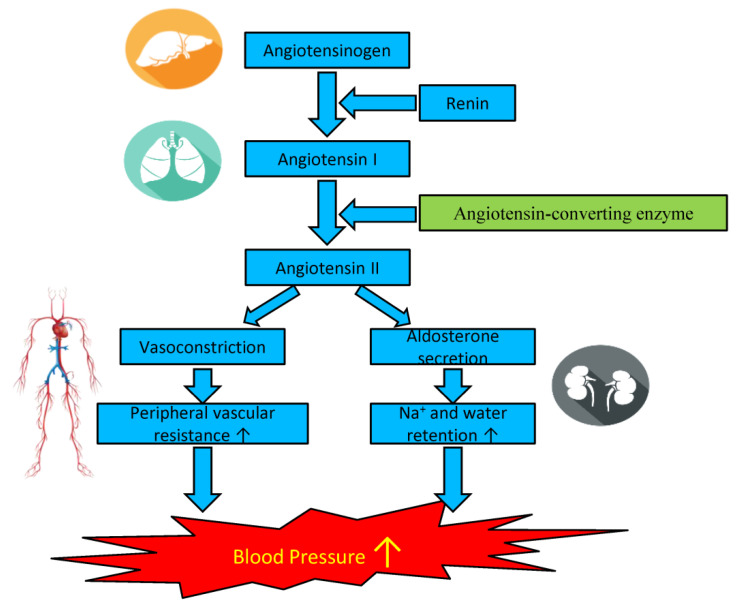
The renin-angiotensin-aldosterone system (RAAS) in the regulation of blood pressure and the function of angiotensin-converting enzyme (ACE).

Angiotensin-converting enzyme (ACE) is a ubiquitous enzyme in mammalian tissues that is involved in the renin-angiotensin and kinin nitric oxide systems, and blood pressure in humans is regulated by the renin-angiotensin-aldosterone system (RAAS) through two main proteases, renin and ACE (Figure 1) [33]. Briefly, ACE can convert angiotensin I (Ang I) into angiotensin II (Ang II) and increase blood pressure by vasoconstriction, which is considered a useful therapeutic target for the treatment of hypertension [34]. To control angiotensin II production, initially, antihypertensive drugs such as captopril, enalapril, lisinopril, and benazepril were synthesized [35,36,37,38]. ACE inhibitors are employed in conditions such as hypertension, heart failure, and diabetes due to their ability to reduce angiotensin II levels, vasoconstriction, aldosterone secretion, and bradykinin [39,40,41]. However, these synthetic drugs usually cause side effects such as erectile dysfunction, persistent dry cough, angioedema, and congenital malformations [33]. Therefore, exploration of antihypertensive drugs has led to the discovery of healthier antihypertensive substances to relieve blood pressure, and an increasing number of ACE inhibitor-like natural compounds, especially biopeptides, have been found in foods such as milk, eggs, meat, fish, soybeans, and their derivatives [42]. These biopeptides show low/no toxicity or no side effects [43]. As promising functional food supplements, microalgae have also received increasing attention due to their high content of valuable natural compounds, and some of these compounds have been applied in the pharmaceutical, cosmetic, and nutraceutical industries [44]. In addition, microalgal hydrolysates, extracts, and biopeptides in particular have shown ACE inhibitory effects and gained increasing attention [45].

The first study of the antihypertensive effects of microalgae was conducted in the 1980s, when Miyakoshi et al., reported that *Chlorella* decreased blood pressure by modulating the RAAS [46,47]. *Chlorella* was also shown to decrease human blood pressure after ingestion [48]. Suetsuna et al., was the first to find that biopeptides from *Chlorella vulgaris* (*C. vulgaris*) and *Spirulina platensis* (*S. platensis*) showed marked antihypertensive effects on spontaneously hypertensive rats (SHRs) [49]. After these findings, through in vivo and in vitro studies, an increasing number of biopeptides from microalgal hydrolysates from the genera *Chlorella*, *Spirulina*, *Isochrysis,* and *Nannochloropsis* were found to show ACE inhibitory effects with functions similar to those of conventional antihypertensive drugs [50,51,52,53]. Moreover, these peptides are often stable in the mammalian digestive system [54]. Since the isolation and identification of ACE inhibitory peptides from microalgae, their molecular mechanisms, such as those determined from molecular docking, and antioxidative properties have emerged [55,56,57]. The relationship between the structure and activity of ACE inhibitory peptides is important for the design of novel drugs [58]. Such a method is also widely used to screen for food-derived peptides and illustrate the biological mechanisms of their functions or activities [59]. The simulation of docking can aid in elaborating the interaction mechanisms of bioactive peptides with receptors from the binding sites and binding types between the receptor and ligands, and the apparent advantages of in silico studies are the time savings and low cost. Some studies on the structural model were also supported by traditional methods such as fluorescence and circular dichroism (CD) spectra, isothermal titration calorimetry, surface plasmon resonance and bio-layer interferometry, and the computational approaches can significantly aid in the study of the bioactive mechanisms of bioactive peptides [60,61,62]. Coincidentally, the molecular docking mechanism of microalgal ACE inhibitory peptides also emerged in recent years. Thus, this review attempts to review the current research related to the antihypertensive effects, ACE inhibitory effects, and molecular docking mechanisms of peptides from microalgae. The keywords “microalgae OR microalgal”, “antihypertensive OR antihypertension”, “peptides”, and “molecular docking” were searched in the Web of Science and Scopus.

## 2. The Relationship between the Primary Structure of Microalgal Peptides and the ACE Inhibitory Effect

**Figure 2:**
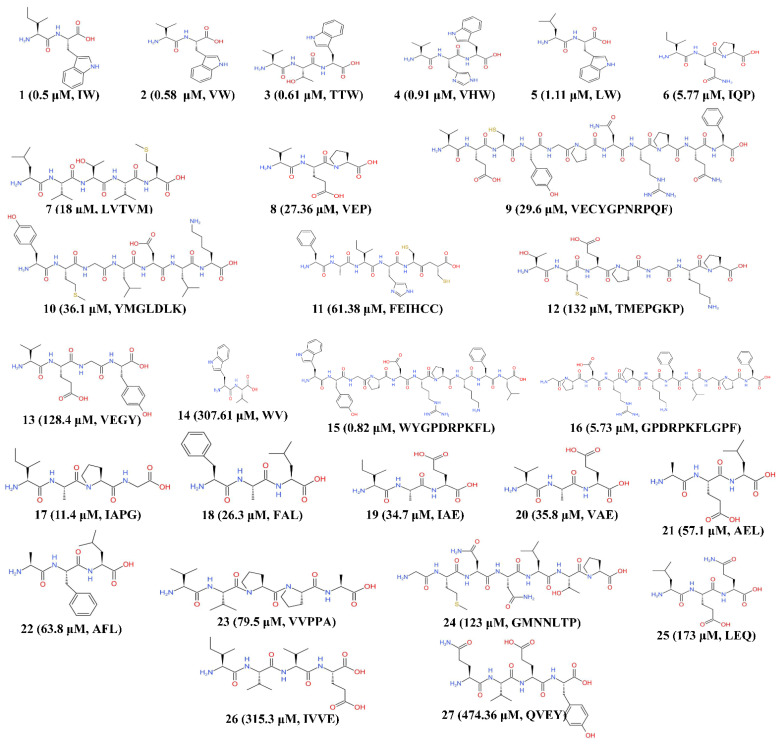
The structural formulas of microalgal ACE-inhibitory peptides. The number of each peptide is consistent with Table 1. The IC_50_ value and amino acid sequence are provided in parentheses.

ACE inhibitor and ACE inhibitor-like peptides could inhibit ACE through two strategies, namely, competitive and noncompetitive, which are determined by Lineweaver–Burk plots. Competitive inhibitors compete with the substrate and bind to the active site of ACE, and noncompetitive inhibitors change the conformation of ACE, which prevents the substrate from binding to the active site of ACE. When the Lineweaver-Burk plot cannot demonstrate the inhibitory strategy of the peptide, a mixed-noncompetitive pattern appears, which has been observed for some peptides [63,64,65]. Notably, the inhibitory activities of ACE inhibitors are not determined by their binding strategy [66]. The ACE inhibitory activity of peptides is expressed as the half maximal inhibitory concentration (IC_50_), which indicates the ACE inhibitor concentration that leads to 50% inhibition of ACE activity. Moreover, a few studies have demonstrated that the composition of the peptide and the amino acid sequence impacts the inhibitory activity of hypertensive peptides [67]. Thus, we listed the microalgal bioactive peptides, their origin, and their IC_50_ values in Table 1; their primary structures are shown in Figure 2.

Daskaya-Dikmen et al., noted that potent ACE inhibitory peptides are generally short-chain peptides consisting of 2–12 amino acids [76]. To the best of our knowledge, the number of amino acids in peptides with antihypertensive properties from microalgae ranges from 2 to 11. To ensure the stability and bioactivity of ACE inhibitory peptides in mammalian gastrointestinal tissue, these peptides are usually obtained from the biomass of microalgal hydrolysates through hydrolytic enzymes under acidic conditions to simulate gastrointestinal digestion. Thus, the peptides identified from microalgae were regarded to be stable in the mammalian gastrointestinal tract.

A few in vitro and in vivo studies have reported IC_50_ values of microalgal ACE inhibitory peptides ranging from 0.5 to 474.36 μM, and most of these microalgal peptides were clearly noncompetitive ACE inhibitors (Table 1), as the noncompetitive pattern seemed to be more common and more effective than the competitive pattern. Except for the peptides with unavailable patterns, the IC_50_ values of competitive peptide inhibitors from *C. sorokiniana* and *C. ellipsoidea* were 307.61 and 128.4 μM, respectively, while most noncompetitive inhibitors from microalgae had lower IC_50_ values, with the exception of peptide Thr-Met-Glu-Pro-Gly-Lys-Pro (TMEPGKP) from *Spirulina sp.* (IC_50_ = 132 μM; the IC_50_ of others ranged from 0.5–61.38 μM). Pujiastuti et al., pointed out that there is no correlation between ACE inhibitory activity and the inhibitory pattern in marine organisms (mainly multicellular organisms such as fish, sharks, and shrimp) [66]. When focusing on only microalgal biopeptides, different inhibitory mechanisms were observed. Such a finding may be a misjudgment because limited research has been conducted on microalgal ACE inhibitory peptides, and more research is needed on microalgal biopeptides to verify the hypothesis that differences in species or genetics may dominate the inhibitory pattern and bioactivity. For instance, to the best of our knowledge, ACE inhibitor-like peptides in the genera *Isochrysis*, *Spirulina,* and *Nannochloropsis* have shown only noncompetitive properties in studies that have analyzed this pattern to date, and *Chlorella* shows both inhibition patterns (Table 1).

The primary structure of the peptide (chain composition, position, and sequence of amino acids) is also an important factor associated with ACE inhibitory activity. The N-terminus and C-terminus both strongly affect the ACE inhibitory activity of biopeptides [41]. Many short-chain peptides with hydrophobic and other amino acids (P, Y, F, or W) at the C-terminus are considered potent ACE inhibitory peptides [77]. In microalgal biopeptides, the residues C, F, L, V, W, P, E, G, A, Q, Y, K, and M at the C-terminus all exhibited ACE inhibitory-like properties to a greater extent than peptides from commercially available foods [41] but to a lesser extent than cereal protein peptides [78] and marine organisms [66].

To better understand the correlation between the structural ACE inhibitory activity from the C-terminal residues in microalgal biopeptides, the structural formulas of these amino acids are shown in Figure 2. F, W, G, and M at the C-terminus of short-chain peptides exhibit relatively strong inhibitory activity against ACE. For instance, when W was the C-terminal residue of microalgal peptides, these microalgal peptides (numbers **1**–**5**) showed low IC_50_ values (0.5–1.11 μM), indicating a strong inhibitory effect, while W at the N-terminus of a dipeptide (number **4**) showed a relatively high IC_50_ value (307.61 μM). In addition, Y as the C-terminal residue of the microalgal peptides did not have strong inhibitory effects [73,75].

In addition to the amino acids at the N- and C-termini, the type of functional group at these positions affects the ACE inhibitory effects of microalgal biopeptides. Hydrophobic and aromatic residues at the N- and C-termini usually show strong antihypertensive activity [79]. Notably, microalgal peptides showed a strong ACE inhibitory effect when short-chain alkyl residues were present at the N-terminus and C-terminus, such as peptides **1**, **2**, **3**, **4**, **5**, and **8**. It may be inferred that the bioactivities of peptides **1**, **2,** and **5** are influenced by their crystal structures, which showed short-chain alkyl groups or a certain number of carbon atoms, whereby the isobutyl group showed the strongest inhibitory activity and *n*-butyl showed the lowest inhibitory activity. The different bioactivities among peptides with similar termini indicated that the other amino acid residues also affect the biofunctions and efficacy against ACE. For example, some residues or atoms can form hydrogen bonds (oxygen-, nitrogen-, benzene-, phenol- and amine-containing residues) [80,81].

## 3. Molecular Docking of Microalgal Peptides to ACE

Great efforts have been made in recent years in the molecular docking of biopeptides from conventional foods to ACE [82,83]. However, unlike ACE inhibitor-like peptides from traditional food sources, the molecular interaction mechanism and molecular docking of microalgal peptides are still untapped. The crystal structure often chosen is human tACE (PDB ID: 1O8A). The best pose and conformation of the peptide and ACE can be determined by LibDock scores and binding energies. LibDock scores are obtained from the IC_50_ value with the formula LibDock score = 10.063 lg (1/IC_50_) + 68.08 [84], and a lower binding energy is better.

**Figure 3:**
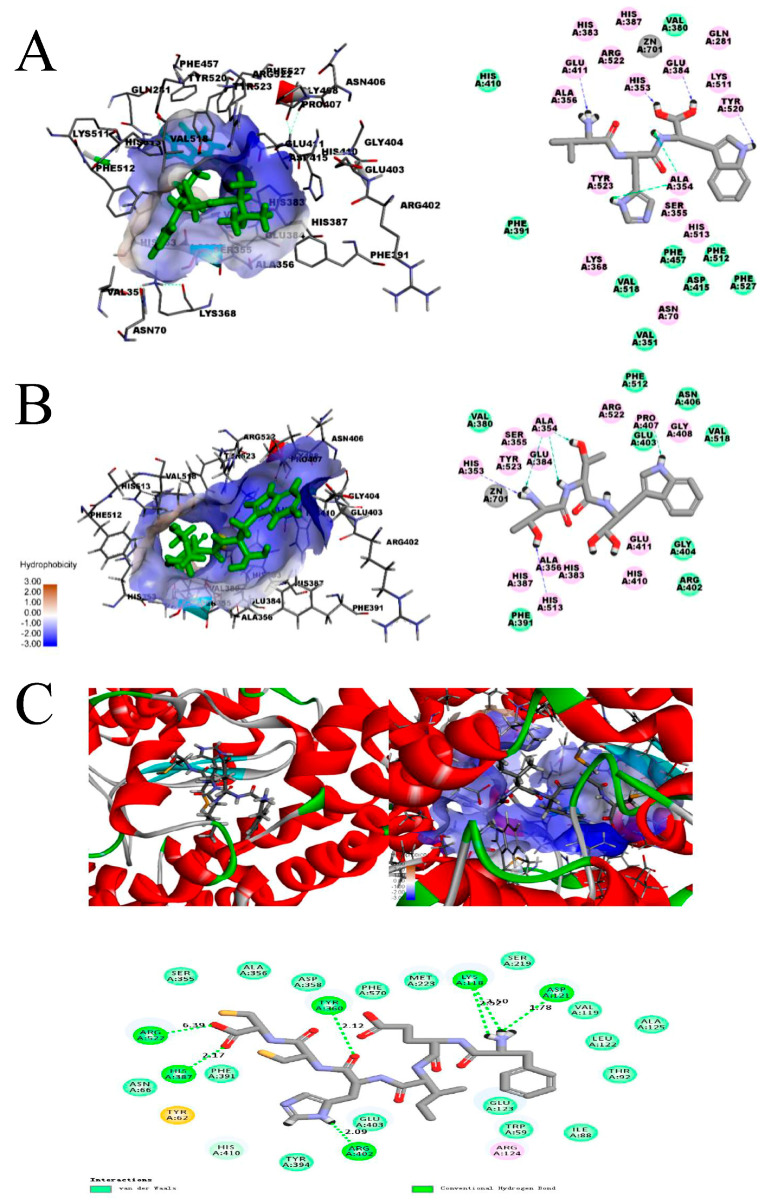
Molecular docking of ACE and the peptides. Docking pose, hydrophobic interaction diagram with active pocket, and two-dimensional diagram of TTW (Thr-Thr-Trp, **A**), VHW (Val-His-Trp, **B**), and FEIHCC (Phe-Glu-Ile-His-Cys-Cys, **C**) binding with ACE, respectively. The dotted lines represent hydrogen bonds, the atoms in green indicate van der Waals interaction forces in (**A**,**B**), and the atoms in light green in (**C**) represent the van der Waals interaction force. The pink atoms indicate an electrostatic interaction force, and gray atoms represent zinc in (**A**,**B**). The difference density map (blue clouds) is the electron cloud of hydrophobic interactions.

Human tACE includes three active site pockets named S1, S2, and S1′ [85]. These pockets contain different amino acid residues: A354, E384, and Y523 in S1; Q281, H353, K511, H513, and Y520 in S2; and E162 in S1′ [75,86]. In addition, these active site pockets have different favorable amino acid residues that bind in them. The S1 pocket shows strong affinity to P, A, V, and L; Pro and Leu are the most favorable for S2 binding; and S1′ is more likely to bind I [87]. In addition, molecular interactions such as van der Waals forces, coordination interactions, hydrogen bonds, and electrostatic, hydrophobic, and hydrophilic forces should be considered. Peptides and ACE residues are linked through the main interaction forces involving van der Waals forces and some secondary interactions, such as hydrogen bonds and hydrophobic and electrostatic forces (Figure 3 [55]).

Even though these forces can contribute during molecular docking, the hydrogen bond is still considered the most important factor in binding stability [88]. The distance of hydrogen bonds between the amino acid residues of ACE and the peptide could reflect the affinity of the peptide. A shorter distance indicates a stronger affinity and more stable binding [89]. Considering the active sites in ACE, the hydrogen bonds between the amino acid residues in the microalgal peptides and the active sites of ACE affect their inhibitory activities and cause discrepancies. The microalgal peptide Thr-Thr-Trp (TTW) (LibDock score: 162) from *C. vulgaris* forms five short hydrogen bonds within the active sites of ACE (ranging from 2.08 to 2.48 Å), which include hydrogen bonds with H353 and H513 in the S2 pocket and A354 in the S1 pocket (Table 2). Moreover, Val-His-Trp (VHW) (LibDock score: 177) can form five short hydrogen bonds with ACE (ranging from 1.93 to 2.45 Å), which include hydrogen bonds with A354 and E384 in the S1 pocket and H353 and Y520 in the S2 pocket. These two peptides can finally construct stable conformations with ACE (Figure 3A,B and Table 2 [55]). However, two points should be noted: (1) these two peptides (TTW and VHW) form hydrogen bonds with P407 and E411, respectively, which are residues that do not belong to well-known active sites in ACE; and (2) E384 in the S1 pocket can coordinate with Zn^2+^, which contributes to the ACE inhibitory activity.

Another molecular docking analysis of ACE with the ACE inhibitory peptide Phe-Glu-Ile-His-Cys-Cys (FEIHCC) from *I. zhanjiangensis* indicated that the microalgal peptide could interact with ACE through hydrogen bonds in sites other than the active sites to have an inhibitory effect on ACE (Figure 3C [51]). However, the inhibitory activity was still affected by whether the peptide formed hydrogen bonds with the active site because the IC_50_ of FEIHCC (61.38 μM) is much higher than that of TTW (0.61 μM) and VHW (0.91 μM), even though FEIHCC formed more hydrogen bonds with ACE (seven hydrogen bonds with distances ranging from 1.78 to 6.39 Å, shown in Table 2). Moreover, hydrogen bonds between ACE and microalgal peptides usually occur with the N- and/or C-terminus, and this result could verify this theory based on their primary structures as mentioned above. In addition, short hydrogen bonds existed in the middle of these peptides. The oxygen and nitrogen atoms in the middle of TTW and VHW could form short hydrogen bonds with A354 in the S1 pocket.

## 4. The Antioxidant and Anti-Inflammatory Properties of Microalgal ACE Inhibitory Peptides against Hypertension

Oxidative stress has been identified as a key etiological factor in the development of hypertension [90]. Some bioactive peptides possessing ACE inhibitory activity from microalgae also display antioxidant activity [54,91]. This is generally because peptides with antioxidant properties are thought to contribute either synergistically or independently to the antihypertensive effects [92]. ACE inhibitory protein hydrolysates from *B. malleus* increased the 2,2-diphenyl-1-picrylhydrazyl (DPPH) free radical scavenging activity and metal chelation ability at a concentration of 2 mg/mL [93]. The ACE inhibitory peptide IQP from *S. platensis* at a concentration of 6.23 mg/mL exhibited a 75.72% radical scavenging activity (RSA) percentage, which reflects the DPPH radical scavenging effects [94]. The peptide Val-Glu-Cys-Tyr-Gly-Pro-Asn-Arg-Pro-Gln-Phe (VECYGPNRPQF) from *C. vulgaris* could effectively scavenge superoxide radicals (IC_50_ of 7.5 ± 0.12 μM); the IC_50_ value of the hydroxyl radical scavenging effect was 8.3 ± 0.15 μM; and the DPPH radical scavenging activity could reach more than 40% at a concentration of 60 μM [91]. A peptidomic study also showed that certain ACE inhibitory peptides from *Tetradesmus obliquus*, such as Ala-Asp-Val-Pro-Phe-Arg (ADVPFR), Ser-Gly-Ser-Trp-Asp-Gly-Thr-Leu-Arg (SGSWDGTLR), Gly-Pro-Lys-Asp-Asp-Pro-Ala-Ala-Trp (GPKDDPAAW), Ser-Trp-Asp-Gly-Thr-Leu-Arg (SWDGTLR), and Ser-Trp-Ile-Ala-Arg (SWIAR), showed antioxidant properties according to their DPPH scavenging activity [74].

The inflammatory processes are associated with hypertension and participate in its development and maintenance [95]. Inflammatory responses also cause endothelial dysfunction and activate endothelial damage and apoptosis [96]. Peptides from microalgae can also regulate some pathways related to inflammation and oxidative stress. The excellent work of Chen et al., revealed that the peptide FEIHCC from *I. zhanjiangensis* inhibits expression of the NF-κB, MAPK, and Akt signaling pathways to block inflammation and endothelial cell apoptosis after Ang II treatment and activates the Nrf2 signaling pathway [51]. In detail, the NF-κB inhibition could be beneficial in treating inflammatory diseases [97], and FEIHCC could inhibit the NF-κB pathway by protecting IκBα degradation and downregulating NF-κB expression and nuclear transport by inhibiting NF-κB DNA binding activity. The downregulation of MAPK expression also indicated that such peptides could mitigate cellular stress or inflammatory cytokines to some degree. The Akt signaling pathway could be regulated to a normal level corresponding to that resulting from Ang II treatment, and overexpression of the Akt pathway may enhance angiogenesis and lead to hepatic portal hypertension [98]. The activation of Nrf2 also mediated endothelial dysfunction, improved endothelial cell activity, and ameliorated mitochondrial and cellular injury [99,100]. It reduced inflammatory cytokine expression (NO, COX-2, and ICAM-1), inhibited the secretion of inflammatory mediators, and decreased the risk of hypertension.

## 5. Antihypertensive Effects of Microalgal Biopeptides

Microalgal biopeptides could be potential sources of ACE inhibitors [66]. The antihypertensive effects and kinetics are different and determined by the species and peptide. For instance, a single dose (200 mg/kg) of the peptidic fraction of *C. vulgaris* significantly reduced the systolic blood pressure of SHRs from 1 to 4 h, and the highest antihypertensive effect appeared at 1 h (a decrease of 49.9 mmHg). This antihypertensive effect continued for 4 h after oral administration, and the highest effect was comparable to that of the captopril group (10 mg/kg) [49]. A 30 mg/kg dose of the freeze-dried biomass of transplastomic *Chlamydomonas reinhardtii* (*C. reinhardtii*) significantly reduced the systolic blood pressure of SHRs after intragastric administration (metal cannula), which contained more than 29% of the peptide Val-Leu-Pro-Val-Pro (VLPVP) [101]. The antihypertensive effects of the recombinant protein from *C. reinhardtii* were demonstrated after intragastric administration of the genetically modified strain of SHRs at a dose of 10 mg of recombinant *AHP3* (gene from the transplastomic strain; complete name: antihypertensive peptides 3) protein per kg of body weight; the maximal decrease in blood pressure was observed 6 h post-administration [56]. The peptide VHW achieved a 31 mmHg systolic blood pressure drop at the end of the experiment, whereas lisinopril achieved a drop of only 10 mmHg and TTW caused a maximal decrease in systolic blood pressure from 239 to 204 mmHg at 2 h (*p* > 0.05). Both TTW and VHW from *C. vulgaris* also influenced diastolic blood pressure (DBP), with TTW leading to a significant reduction in DBP from 180 to 140 mmHg at 2 h (*p* < 0.05) and VHW showing a decrease in DBP from 174 to 153 mmHg at 1 h (*p* > 0.05) in SHRs at a dose of 5 mg/kg body weight, where these peptides were digested in vitro and administered by gavage [55]. Protein hydrolysates from *Bellerochea malleus* (*B. malleus*) were found to reduce blood pressure in SHRs by 17 mmHg after 5 days of oral administration [93]. These findings indicate that proper application of microalgal peptides has potential protective and therapeutic effects against hypertension.

## 6. Conclusions

Microalgae have great potential for the prevention and treatment of hypertension due to excellent biological properties; they are easily cultured, possess low/no toxicity, and do not require land use. Some have found applications in many health-related industries. Microalgal peptides are effective ACE inhibitor peptides both in vivo and in vitro. The ACE inhibitory effects of these peptides are becoming a popular research topic, as are ACE inhibitor peptides from other food sources. Microalgal peptide activity may be determined by many factors, such as the species of origin, inhibitory pattern, sequence of amino acids, functional groups present, crystal structure, and molecular docking sites. In addition, these peptides protect cells by improving their antioxidant and anti-inflammatory properties through the regulation of physiological signaling pathways such as NF-κB, Nrf2, MAPK, and Akt, which also contribute to the prevention, diagnosis, and treatment of hypertension and its syndrome. However, knowledge of the structure-activity relationships of microalgal peptides is still deficient, especially regarding contributions from other functional groups. Future studies should focus on the bioactivity of microalgal peptides, the diversity of microalgal biopeptides, the relationship between their higher-order structure and activity, and additional mechanisms of molecular docking and interactions to achieve a better understanding of their use in healthy food supplemental sources and drug design.

## Figures and Tables

**Table 1:** The ACE inhibitor-like peptides from microalgae and their inhibitory effects.

Inhibitory Pattern	Species	No.	Peptides Sequence	IC_50_ (μM)	Study Type	Reference
Noncompetitive	*Chlorella sorokiniana*	**1**	IW	0.5	In vitro (rabbit lung)	[68]
		**2**	VW	0.58	In vitro (rabbit lung)	
	*Chlorella vulgaris*	**3**	TTW	0.61	In vivo (rats)	[55]
		**4**	VHW	0.91	In vivo (rats)	
	*Chlorella sorokiniana*	**5**	LW	1.11	In vitro (rabbit lung)	[68]
	*Spirulina platensis*	**6**	IQP	5.77	In vivo (rats)	[69]
	*Nannochloropsis oculata*	**7**	LVTVM	18	In vitro	[70]
	*Spirulina platensis*	**8**	VEP	27.36	In vivo (rats)	[71]
	*Chlorella vulgaris*	**9**	VECYGPNRPQF	29.6	In vitro	[54]
	*Isochrysis galbana*	**10**	YMGLDLK	36.1	In vitro	[72]
	*Isochrysis zhanjiangensis*	**11**	FEIHCC	61.38	In vitro	[51]
	*Spirulina sp.*	**12**	TMEPGKP	132	In vitro	[52]
Competitive	*Chlorella ellipsoidea*	**13**	VEGY	128.4	In vivo (rats)	[73]
	*Tetradesmus obliquus*	**14**	WV	307.61	In vitro (rabbit lung)	[68]
Not available	*Tetradesmus obliquus*	**15**	WYGPDRPKFL	0.82	In vitro	[74]
		**16**	GPDRPKFLGPF	5.73	In vitro	
	*Spirulina platensis*	**17**	IAPG	11.4	In vivo (rats)	[49]
	*Chlorella vulgaris*	**18**	FAL	26.3	In vivo (rats)	
	*Spirulina platensis*	**19**	IAE	34.7	In vivo (rats)	
		**20**	VAE	35.8	In vivo (rats)	
	*Chlorella vulgaris*	**21**	AEL	57.1	In vivo (rats)	
		**22**	AFL	63.8	In vivo (rats)	
		**23**	VVPPA	79.5	In vivo (rats)	
	*Nannochloropsis oculata*	**24**	GMNNLTP	123	In vitro	[53]
		**25**	LEQ	173	In vitro	
	*Chlorella vulgaris*	**26**	IVVE	315.3	In vivo (rats)	[49]
	*Gracilariopsis lemaneiformis*	**27**	QVEY	474.36	In vitro	[75]

Abbreviations of amino acids: A (Ala), R (Arg), N (Asn), D (Asp), C (Cys), Q (Gln), E (Glu), G (Gly), H (His), I (Ile), L (Leu), K (Lys), M (Met), F (Phe), P (Pro), T (Thr), W (Trp), Y (Tyr), V (Val).

**Table 2:** Distances and binding sites of hydrogen bonds for each peptide.

Peptides	Hydrogen Bond	Distance (Å)	Active Sites Pockets
TTW	His353	2.17	S2
	Ala354	2.08	S1
	His513	2.48	S2
	Ala354	2.02	S1
	Ala354	2.46	S1
	Pro407	1.89	-
VHW	Glu411	2.08	-
	Ala354	2.41	S1
	Ala354	1.96	S1
	His353	2.45	S2
	Tyr520	2.23	S2
	Glu384	1.93	S1
FEHICC	Arg522	6.39	-
	His387	2.17	-
	Arg402	2.09	-
	Tyr360	2.12	-
	Lys118	2.52	-
		2.50	
	Asp121	1.78	-
